# Logistical, technical, and radiation safety aspects of establishing a radiopharmaceutical therapy program: A case in Lutetium‐177 prostate‐specific membrane antigen (PSMA) therapy

**DOI:** 10.1002/acm2.13899

**Published:** 2023-01-13

**Authors:** Jacqueline E. Zoberi, Jose Garcia‐Ramirez, David Luechtefeld, Nichole M. Maughan, Maxwell Amurao, Reiko Oyama, Brian C. Baumann, Hiram A. Gay, Jeff M. Michalski

**Affiliations:** ^1^ Department of Radiation Oncology Washington University School of Medicine Saint Louis Missouri USA; ^2^ Environmental Health and Safety Washington University School of Medicine Saint Louis Missouri USA; ^3^ MIR Cyclotron Facility and Nuclear Pharmacy Washington University School of Medicine Saint Louis Missouri USA

**Keywords:** commissioning, QA/QC, radiopharmaceutical therapy, safety

## Abstract

Prostate‐specific membrane antigen (PSMA) is a cell surface protein highly expressed in nearly all prostate cancers, with restricted expression in some normal tissues. The differential expression of PSMA from tumor to non‐tumor tissue has resulted in the investigation of numerous targeting strategies for therapy of patients with metastatic prostate cancer. In March of 2022, the FDA granted approval for the use of lutetium‐177 PSMA‐617 (Lu‐177‐PSMA‐617) for patients with PSMA‐positive metastatic castration‐resistant prostate cancer (mCRPC) who have been treated with androgen receptor pathway inhibition and taxane‐based chemotherapy. Therefore, the use of Lu‐177‐PSMA‐617 is expected to increase and become more widespread. Herein, we describe logistical, technical, and radiation safety considerations for implementing a radiopharmaceutical therapy program, with particular focus on the development of operating procedures for therapeutic administrations. Major steps for a center in the U.S. to implement a new radiopharmaceutical therapy (RPT) program are listed below, and then demonstrated in greater detail via examples for Lu‐177‐PSMA‐617 therapy.

## 1 INTRODUCTION

Radiopharmaceutical therapy (RPT) is a form of internal radiation therapy using unsealed radionuclides to emit energetic particles (beta, Auger, alpha) in close proximity to tumor cells for dose deposition. For some therapies, these radionuclides are joined to molecules to target the delivery of the radiation on the basis of specific biologic features to maximize dose to the tumor while sparing normal tissues. Prostate‐specific membrane antigen (PSMA) is a cell surface protein highly expressed in nearly all prostate cancers, with restricted expression in some normal tissues such as the small intestine, proximal renal tubules, and salivary glands.^1^ PSMA‐617 is a small molecule which shows a high binding affinity to PSMA and efficient internalization and retention with the cancer cells.^1^ PSMA‐617 radio‐labeled with lutetium‐177 (Lu‐177) enables PSMA‐targeted radioligand therapy. Lu‐177 has a physical half‐life of 6.7 days and undergoes beta decay emitting beta particles with maximum energies (abundances) of 497 keV (78.6%), 384 keV (9.1%), 176 keV (12.2%) and low energy gamma photons with energies (abundances) of 208 keV (11%) and 113 keV (6.6%).^2^ The beta particles deposit most of their energy in tissue to less than 2 mm (mean penetration of less than 1 mm), and the gamma rays are generally useful for imaging the biodistribution.^2^, ^3^, ^4^, ^5^ Biologic clearance of the non‐tumor bound radioligand is via the kidneys and is mostly complete by 48 h.^3^, ^5^

Multiple randomized trials of targeted radionuclide therapy using Lu‐177‐PSMA‐617 have reported positive results in patients with metastatic castration‐resistant prostate cancer (mCRPC), most notably the VISION trial for patients treated with androgen receptor pathway inhibition and taxane‐based chemotherapy,^6^, ^7^, ^8^ with more prospective clinical trials evaluating Lu‐177‐PSMA‐617 and other PSMA‐targeted ligands for other patient populations, including pre‐taxane and hormone‐sensitive patients, underway.^9^, ^10^ In March of 2022, based on the results of the VISION trial, the FDA (Food and Drug Administration) granted approval of the use of Lu‐177‐PSMA‐617 for patients with mCRPC who test positive for PSMA with an FDA‐approved imaging diagnostic agent and have been treated with androgen receptor pathway inhibition and taxane‐based chemotherapy.^11^ Therefore, the use of Lu‐177‐PSMA‐617 is expected to increase and become more widespread. Recent articles have summarized aspects of Lu‐177 PSMA therapy.^9^, ^10^, ^12^ Here, we describe the logistical, technical, and radiation safety considerations for implementing a RPT program, with particular focus on the development of operating procedures for therapeutic administrations. An outline of major tasks for a center in the U.S. to implement a new RPT program is shown below, and each task is then demonstrated in greater detail via examples for Lu‐177‐PSMA‐617 therapy.

Implementation Tasklist:

1. Learn about the therapy
2. Allocate resources
  - Staffing
  - Equipment
  - Space
3. Satisfy radioactive material (RAM) use regulations
4. Generate the written procedures and accompanying forms
  - Patient eligibility, schedule coordination, and informed consent
  - Logistical, technical, and radiation safety aspects of the source/drug
    - Description of source, drug, and treatment schema
    - Drug storage & handling
    - Drug ordering, manufacturing, shipping & receiving
    - Drug manipulation/dispensation, assay, quality control checks
      - Schedule test shipment for a calibration vial of drug to calibrate measurement equipment
      - Use test shipment as a dry run to validate/fine‐tune procedures and forms (everything besides treatment infusion)
      - Use test shipment to assess exposure levels in and around treatment area (and restroom)
  - Overall therapy administration workflow
    - Pre‐therapy preparation (AFTER identifying a treatment area/restroom)
      - Preparing treatment area/restroom for contamination control
      - Preparing the patient (both medical and radiation‐wise)
      - Preparing the treatment staff
    - Therapy administration
      - Infusion method—vial‐ or syringe‐based
      - Extravasation
    - Post‐therapy procedures
      - Determination of administered activity
      - Patient monitoring
      - Patient release determination and radiation safety instructions
      - Customization of release instructions for special cases
      - Handling of other situations after release, for example, medical emergencies, lab samples, patient expiration
      - Contamination checks and area release
  - Generate (or supply) forms—including, but not limited to
    - Provide example of a calibration document from the manufacturer
    - Study‐specific forms, if applicable
    - Dosage dispensation form and activity labels (if drawing performed)
    - Activity determination form
    - Written directive (i.e., RAM prescription form)
    - Administered activity, survey, and release documentation
    - Radiation warning/Restricted area signage for the treatment area/restroom
    - Restroom etiquette instructions for posting in the restroom
    - Checklists for pre‐treatment preparation and for infusion
    - Radiation safety/discharge instructions
    - Radioactive patient identification card
5. Conduct Training
  - Written procedures
  - Radiation safety
  - Mock infusion session(s) with non‐RAM solution
  - Kick‐off meeting
6. Initiate and Maintain Therapy
  - Initiate and provide on‐going support of the therapy
  - Keep procedures and forms up to date
  - Maintain records
  - Modify the SOP in response to problems or events

## 2 LEARN ABOUT THE THERAPY

The center first needs to gather information and learn about the therapy. Key things to learn include the prescribing information, the pharmacokinetic profile of the drug, its form and handling requirements, and its radiation properties. Typically, such information is documented and readily available from either a study sponsor or radiopharmaceutical drug company, depending on whether the drug is administered in a clinical trial or off‐trial as standard‐of‐care. Other key information may not be as readily available, for example, the drug manufacturing and shipping schedule, how the drug activity is calibrated, how the drug is prepared for administration, how the drug is administered, how to manage radioactive contamination and waste, and how to manage radioactive patients. For these aspects, it may require a more active gathering of information by reaching out to someone familiar with drug manufacturing, and reaching out to other centers that have experience with the drug therapy.

## 3 ALLOCATE RESOURCES (STAFFING, EQUIPMENT, SPACE)

Staffing allocation starts very early on in the implementation process by identifying members of the team who are needed not just for treatment support but also for discussing the logistics involved in bringing a new therapy to the clinic. Such discussions should also include the study sponsor/drug company, drug manufacturer, and someone on the sponsor/company side familiar with drug administration. While the staffing allocation can depend on the infrastructure at a particular center, key members for drug administration should include a schedule coordinator, prescribing physician (depending on the setting, either a radiation oncologist and/or nuclear medicine physician), medical physicist (therapy or nuclear medicine), technologist (radiation therapist (RTT) or nuclear medicine technologist (NMT)), nurse, and the radiation safety officer (RSO). Additional radiation safety support may be available from health physicists and environmental health/safety staff. Support may be needed from radiopharmacists if drug manipulation, dispensation, or quality testing is required, and from radiation‐trained inpatient care staff if such care is needed post‐administration of the drug.

Equipment must be acquired for tasks involved with radioactive contamination control (e.g., personal protective equipment, absorbent paper, tape, sealable plastic bags, trash containers, wipes, foaming cleansers, and toilet brushes), for radiation exposure control (e.g., vial/syringe shielding, mobile shields, and long tongs), for drug transport and preparation (e.g., transport container/cart, equipment for dispensation of dose from vial into syringe, if done), as well as for drug administration (e.g., intravenous (IV) starter kits, IV lines, needles, sterile saline, IV pole, infusion pump, administration chair and table). Radiation measurement equipment (e.g., body and extremity radiation dosimeters, survey meters, well counters, and dose calibrators) must be acquired. The equipment must satisfy regulatory requirements^13^ as well as good clinical practice standards for calibration and quality control (QC). The equipment listed above is not intended to be an exhaustive list as the specific equipment needed will be dependent on the particular implementation at the treating center.

Space for radiopharmaceutical and patient handling also needs to be identified, and these designated areas must satisfy regulatory requirements for acceptable levels of ambient radiation exposure and removable contamination. For some therapies, these areas (treatment, restroom, and patient holding) are designated with the understanding that they will need to be restricted areas with limited access and checked for absence of contamination prior to release for normal routine use.

Specific examples of these resources will be outlined below in reference to Lu‐177‐PSMA‐617 therapy.

## 4 SATISFY RADIOACTIVE MATERIAL USE REGULATIONS

The next major step is to work with the radiation safety officer to ensure the RPT program satisfies the applicable regulatory requirements for medical use of radioactive material (RAM), which could either be the federal Nuclear Regulatory Commission (NRC) or a state‐governing body authorized by the NRC and following 10 CFR Part 35.^13^ A RAM license is required for possession and medical use of the radioactive isotope (§35.11, §35.300).^13^ Prescribing physicians need to be identified as Authorized Users (AUs) on the RAM license, which entails satisfaction of training requirements outlined by the NRC (§35.390 and §35.396).^13^ Supporting staff can be listed as “Supervised Individuals” (§35.27) on the RAM license, for example, medical physicists, health physicists, technologists, nurses, who can be designated to perform tasks associated with handling and administration of RAM under the AU's supervision.^13^ The isotope and activity limits, that is, a per‐use quantity and a maximum possession quantity, need to be specified on the RAM license (10 CFR 35.12(b) and NRC Form 313 at nrc.gov). Radiation measurement equipment must meet calibration and QC requirements (§35.60).^13^ Support from radiation safety experts, for example, the radiation safety officer, medical physicists, and health physicists, is key to ensuring regulatory requirements are met by the RPT program. These are just some key requirements as the list here is not meant to be exhaustive. Generally speaking, radiation safety activities should be performed in accordance with regulatory requirements, the license, and licensee‐approved written procedures.

## 5 GENERATE THE WRITTEN PROCEDURES AND ACCOMPANYING FORMS

Per 10 CFR35.41, the licensee shall develop, implement, and maintain “written procedures” that provide a high level of confidence that the treatment is in accordance with the “written directive”, which is essentially a RAM prescription. For unsealed byproduct material, the written directive (§35.40) specifies the patient's name, the radioactive drug, dosage, and route of administration. The written procedure must address these aspects of treatment. The written procedure should also describe that if the treatment is not in accordance with the written directive, then the licensee shall determine whether a medical event has occurred and, if so, follow the steps for reporting, as described in 10 CFR 35.3045.^13^ Beyond this, the level of detail in the written procedure is at the discretion of the licensee, but as an operating procedure it should outline the roles and responsibilities of the team, and outline processes that ensure that handling of RAM is carried out in a manner that satisfies regulatory requirements,^13^, ^14^, ^15^ is safe, and aligns with best practice recommendations.^16^ At our center, the radiation oncology medical physicist takes the lead on writing these operating procedures, which describe the following three aspects:

a. Patient eligibility, schedule coordination, and informed consent
b. Logistical, technical, and radiation safety aspects of the source/drug
c. Overall therapy administration workflow

The procedures for **Task (4a)** (in the Implementation Tasklist) are largely driven by the study sponsor/radiopharmaceutical company and drug manufacturer and will be essential information to the schedule coordinator and AU (perhaps with medical oncology) when selecting and scheduling patients. Procedures should describe the eligibility/screening criteria for patient selection. Based on the VISION trial, eligible patients for treatment with Lu‐177‐PSMA‐617 off‐study will generally be those with castrate‐resistant metastatic prostate cancer who have progressed after taxane‐based chemotherapy and have PSMA‐positive metastatic lesion(s) seen on PSMA PET/CT scan with no PSMA PET‐negative lesions that meet certain size criteria. Description of the acquisition and interpretation of these PET scans are beyond the scope of this article and have been addressed elsewhere.^6^, ^17^ Patients should also have an ECOG performance status of 0–2, a life expectancy greater than 6 months, and adequate organ and bone marrow function.^6^ Patients typically receive follow‐up imaging, including CT, MRI, and bone scans. The acquisition of post‐treatment quantitative SPECT imaging for response assessment and dosimetry may also be done but is not currently standard of care in the United States. For interested readers, published requirements and recommendations for dosimetry are available from Europe.^18^ Thus, RPT not only involves scheduling of the drug administration with the treating staff, but may also require the scheduling of screening tests and imaging procedures with other departments, and the procedures should address how these appointments are scheduled in coordination with the drug administration. The procedures should also describe the drug manufacturing and shipping schedule as this will dictate the order deadline for these drugs, and can limit drug availability and, hence, treatment administration days to certain days/times of the week. It is critical to establish the date (and time) of administration and avoid delays in treatment as the activity of the drug will depend on this date/time, and will decay according to the physical half‐life of the radioisotope. For a single patient receiving RPT, many of these scheduling efforts need to be repeated several times as dosages can be administered in multiple cycles (fractions) separated by lengthy intervals, for example, Lu‐177‐PSMA‐617 is administered in up to six fractions every 6 weeks. Scheduling of the treatment involves communication with many parties, both internal and external to the radiation treatment team, and the communication chain should be clearly established by the coordinator before scheduling the first patient.

The procedures for **T** **ask** **(4a)** should also describe the informed consent process to describe the therapy to the patient and assess patient compliance. The latter may include designing handouts like a “what to expect” or “radiation safety instructions upon discharge.” Some of these documents may already be available from the company/sponsor and can be customized by the center as needed.

The procedures for **T** **ask (4b)** are for those involved with all aspects of drug handling, excluding treatment administration, and describe four aspects of the source/drug, namely:

i. Description of source, drug, and treatment schema
ii. Drug storage and handling
iii. Drug ordering, manufacturing, shipping, and receiving
iv. Drug manipulation/dispensation, assay, and quality control checks

For **T** **asks** **(4b.i.)–(4b.ii)**, it is necessary to refer to any reference materials provided by the sponsor/company. The procedures should describe the drug product and the radioisotope, namely the mechanism of action, the emission spectra, and the pharmacokinetic profile (as described in the Introduction). The procedure should identify the treatment site, the organs at risk, prescription dosage, and fractionation schedule. For Lu‐177‐PSMA‐617, prescribed activity is 7.4 GBq (200 mCi) per fraction. Based on a sub‐study of 29 patients from the VISION trial, the organs with the highest radiation absorbed doses are lacrimal glands, salivary glands, large intestine, kidneys, and urinary bladder wall, as described in the manufacturer‐provided Prescribing Information.^11^ Activities for subsequent fractions can be modified based on adverse reactions (myelosuppression, renal toxicity, dry mouth, gastrointestinal toxicity, etc.). Modifications include delaying the next fraction for Grade 2 reactions until they have reached Grade 1 or baseline, reducing the activity by 20% for Grade 3 reactions, and discontinuing treatment for recurrent Grade 3 or Grade 4 reactions.^11^ The procedures should describe storage and handling requirements, namely the form, volume, thawing requirements if any, storage conditions, shelf‐life, and disposal requirements. Lu‐177‐PSMA‐617 does not require any refrigeration or thawing, as it arrives in a clear, glass vial at room temperature, and can remain this way for five days. With regard to disposal requirements for Lu‐177‐PSMA‐617 most of the waste generated from these therapies can be stored in a dedicated, on‐site decay‐in‐storage (DIS) facility until exposure levels are indistinguishable from background and can be disposed of following the appropriate non‐radioactive waste stream. Direct and indirect reactor production methods can be used to obtain Lu‐177 and depending on which is used, there may also be a Lu‐177m impurity (long‐ lived isomer with T_1/2_ = 160 days) in the radioactive waste that needs to be transferred to a suitable waste disposal facility.^2^ Thus, knowledge of the radioisotope production method for the particular radiopharmaceutical is key to understanding its impact on RAM waste management.

For the next two tasks, **(4b.iii.)–(4b.iv.)**, procedures should be drafted based on information provided by the sponsor/company and with input from the key members involved with these items. Once procedures are drafted, a good opportunity to fine‐tune/validate these procedures is during calibration of measurement equipment, used for activity determination and for drug manipulation/dispensation, for the particular radioisotope and geometry (§35.60b).^13^ In the case of Lu‐177‐PSMA‐617, this would involve receipt of a test shipment of a calibration vial of Lu‐177 containing an amount of activity traceable to a certified metrologic center, assay of this vial in the center's dose calibrator, and configuring the appropriate calibrator settings relating the instrument reading to activity. These steps should be carried out for dose calibrators that will be used to assay Lu‐177‐PSMA‐617 at the treating center because inconsistencies in activity measurements can arise as particles from beta emitters interact with various materials. The mean free path of beta particles is heavily dependent upon the materials they come in contact with, and the resulting Bremsstrahlung radiation must also be considered in different measuring environments. Factors such as the volume of radiopharmaceutical, the geometry, and material composition of the container (vial or syringe, glass or plastic, thickness, etc.) can affect the activity measurement. Per the manufacturer (Advanced Accelerator Applications, AAA, a Novartis Company, Saint‐Genis‐Pouilly, France), standard Lu‐177 sources produced at each manufacturing site are sent to the corresponding Metrologic Center (e.g., the National Physics Laboratory [NPL] in England, the Energia Nucleare ed Energie Alternative [ENEA] in Italy, the National Institute of Standards and Technology [NIST] in the United States, etc.) for activity certification. The standard source is provided by the manufacturer as a calibration vial of Lu‐177 radionuclide in water solution to the treating center and should mimic the packaging and volume of the radiopharmaceutical used for patient dosing to guarantee the same measurement conditions. Measurement of this calibration vial is generally one of the final steps required by the sponsor/company prior to providing drug for clinical use. Thus, this test shipment can also be used as a dry run to see first‐hand how the dosage, packaging, and documentation appear, and then use it to verify aspects of the procedure, namely:

- Verify ordering logistics and timelines.
- Verify shipping route as well as timeline from manufacturer to center.
- Verify entire chain of events at treating center, from drug receipt to waste management, excluding actual infusion of the RAM.
- Prior to any drug manipulation/dispensation, perform survey measurements of the unshielded vial in and surrounding the treatment area/restroom to give worst‐case (maximum) exposure rate estimates (described for **T** **ask (4c)** as part of the commissioning of the treatment area).

Perhaps the most complex portions of the procedure to have ready prior to receipt of the test shipment are those for syringe‐based infusions involving drug manipulation/dispensation and activity assay. The processes, forms, and equipment need to be commissioned prior to receipt of test dosage and the staff responsible for these steps also need to be available for the dry run. Drug manipulation/dispensation into the syringe can be performed at a radiopharmacy or by trained staff on‐site at the treating center. Note that the preparation practice/environment will affect the beyond‐use‐date (BUD) for the drug. For those centers performing dosage dispensation in a hot lab area with ambient or unfiltered air, this would allow for “immediate use” of the drug, meaning drug must be administered within 1 h of exposing the vial contents to the room air, for example, via needle puncture of the vial septum. For those centers with a hot lab area satisfying either USP 797 or 825 standards for cleanrooms, the BUD can be extended.^19^ For example, in our center, nuclear pharmacists perform the dispensation into a syringe using an International Organization for Standardization (ISO) Class 5 Primary Engineering Control (PEC) hood located in an ISO Class 8 area,^19^ yielding a BUD of 24 h. Once the drug is in its final form, the activity can be independently assayed by medical physicists in a separate dose calibrator prior to treatment.

Besides drug manipulation/dispensation and activity determination, the procedures should outline QC checks of the drug prior to administration. QC checks should include verification of correct patient, fraction number, isotope, drug, form/route, and activity amount to ensure that the treatment will be in accordance with the written directive (§35.40).

The procedures for **T** **ask** **(4c)** are for those involved with drug administration (once QC checks of the drug are complete) and patient care. **Task** **(** **4c** **)** should describe the overall therapy administration workflow, and is the largest section of the procedure which can be further broken down into three major sub‐sections, namely:

i. Pre‐therapy preparation procedures
ii. Therapy administration procedures
iii. Post‐therapy procedures

Before describing the procedures for **T** **ask** **(4c)**, the treatment administration area needs to first be identified; its location largely depending on the risks of contamination and of exposure, which are considered here separately. The risk of contamination in or around the treatment area is dependent on the treatment time and nature of the infusion, for example, vial‐ versus syringe‐based. If the treatment time also includes post‐infusion monitoring of the patient, and if there is a high clearance rate of the drug through the urine, then the chance for contamination is higher if the infused patient needs to use a restroom. Because of these contamination risks, it is critical to identify a “restricted” area for treatment/monitoring and a nearby restroom (10 CFR Part 20.1003), that can be isolated from the general traffic by placing barriers and posting radiation warning signage, and, if needed, locked down for decontamination/decay purposes.^14^

**FIGURE 1:**
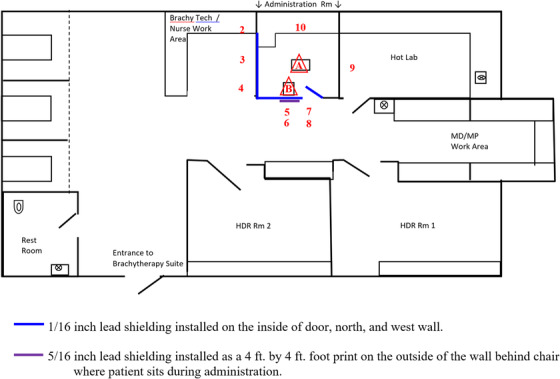
Diagram of administration room (also shown in Figure 2) with the numbered survey locations. The room is situated in the Brachytherapy suite, dedicated restroom as indicated in diagram. Location A indicates where the unshielded calibration vial was located for commissioning survey, and represents where the drug would be situated for infusion. Location B represents where a patient sat during a verification survey, and represents where any patient would be situated for infusion. Dimensions of the administration room are 11.5 ft wide by 8.5 ft deep (not drawn to scale). Please note there is lead shielding in the walls of the room as it was originally designed for sodium iodide iodine‐131 thyroid carcinoma therapy.

**FIGURE 2:**
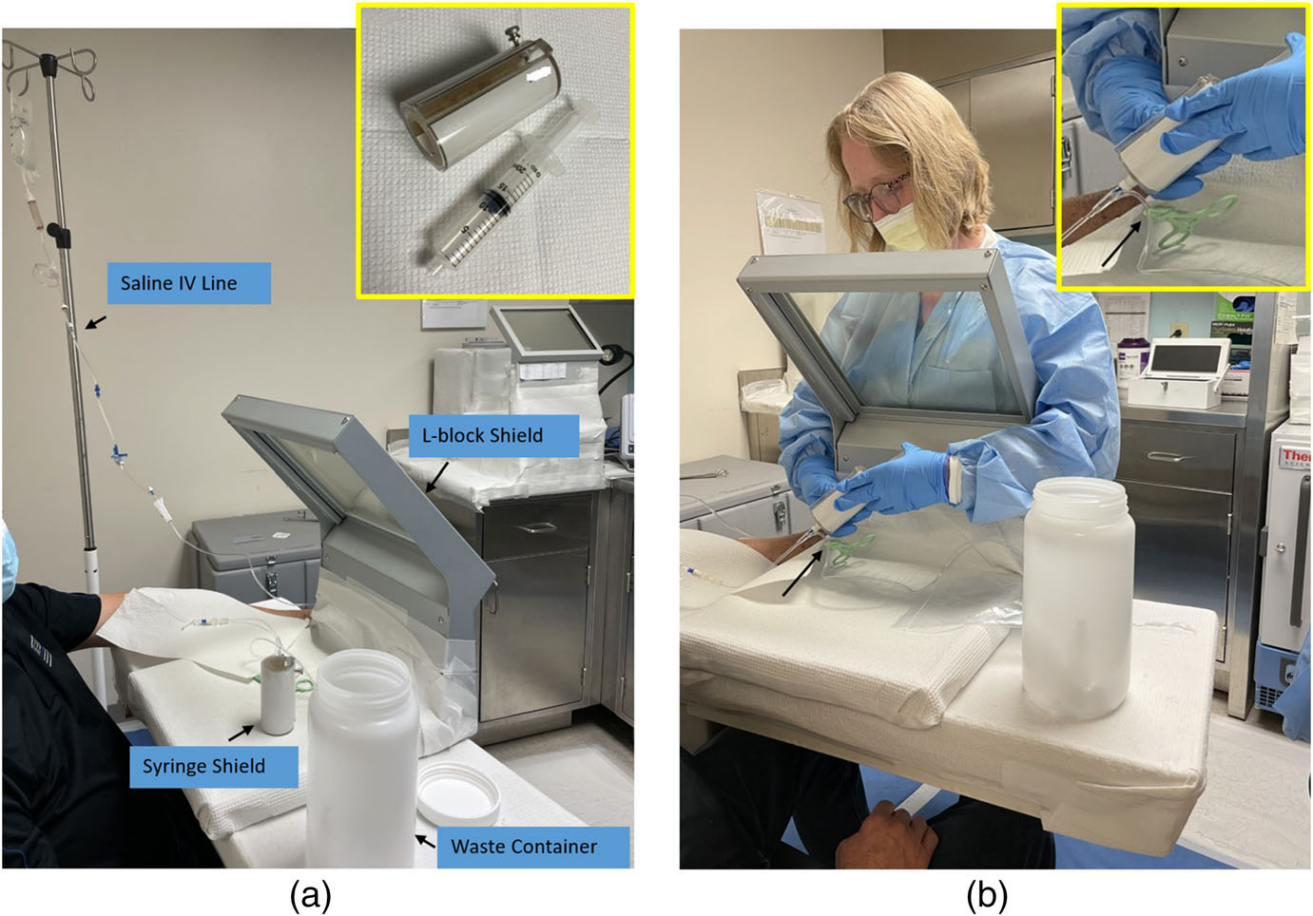
(a) Set‐up for infusion in the administration room. Nursing has inserted an indwelling catheter (IV tubing with y‐connector) in the patient's antecubital vein, and has started fluids. Note covering of surfaces with absorbent paper, with the ability to lift the covering on the patient's arm to check for any extravasation during infusion. Inset shows separately a 20 ml syringe and the syringe shield (composed of a plastic barrel to attenuate beta emission and lead lined to attenuate gamma and errant bremsstrahlung emission). (b) Technologist, donned with PPE and personal radiation dosimeters, administers the drug under supervision by the AU (not shown) via manual push on a 20 ml syringe with syringe shield. Arrow indicates where the saline IV line is clamped while infusing the drug through the patient IV line (inset shows magnified view). There are at least three flushes of the syringe with saline after administration of the drug, involving a series of exchanging the clamp between patient line and saline line. Finger and chest dosimeters measured less than 1 mrem for the administration.

The risk of exposure should also be addressed when deciding on the treatment/monitoring area and dedicated restroom. The risk of exposure in or around these areas is largely dependent on the emission spectra from the radioisotope and levels of activity involved. For any RPT, it is important to keep exposures as low as reasonably achievable (ALARA) by applying the concepts of distance, time, and shielding. Distancing measures may include choosing areas that can be restricted and kept at some distance from public access. Time of exposure is dictated by length of time around the drug and the infused patient, and should be minimized as much as possible. Shielding measures may include the use of lead and/or plastic, depending on the emission type and spectra, and come in different forms, for example, shielded vial containers, syringe shields, L‐block and rolling shields, and wall shielding. As part of commissioning of the treatment area, radiation exposures can be assessed via surveys using the calibration vial prior to first patient treatment. These exposure measurements should be repeated during actual patient treatments to verify safe exposure levels to those in the surrounding area (per NCRP Reports 49,147).^20^, ^21^ In Table 1, we present the survey results using the calibration vial to commission our Lu‐177‐PSMA‐617 administration room (shown in Figure 1). This room was originally constructed with lead wall shielding for treatment of iodine‐131 (I‐131) sodium iodide thyroid carcinoma patients. The half‐value layer of lead for Lu‐177 versus I‐131 is 0.54 mm versus 2.74 mm, respectively; hence, the emissions from Lu‐177 are well‐shielded by this room.^22^ Note the room dimensions are small, and immediately adjacent to other work areas, but with a larger, more isolated room, wall shielding may be unnecessary. In Table 1, we also include the survey results taken to verify safe exposure levels during an actual patient administration using treatment setup and radiation protection measures as displayed in Figure 2. Considering that infusions are less than 10 min, the survey results indicate that exposures in the different locations should be less than 1 mR in any hour.

**TABLE 1:** Survey of administration room at time of commissioning and later at time of verification for 7.4 GBq of Lu‐177

Location #	Location description	Exposure rate (mR/h) with unshielded calibration vial at location A *(commissioning)* ^a^	Exposure rate (mR/h) with patient at location B *(verification)* ^a^
N/A	Background	0.02	0.02
2	1 ft from wall at nurse desk computer	0.20	0.20
3	1 ft from wall at nurse work area	0.20	0.06
4	1 ft from wall at nurse work area	0.15	0.06
5	1 ft from wall at computer workstation chair level	0.04	0.07
6	1 ft from wall at computer workstation standing eye level	0.30	0.13
7	1 ft from open doorway waist level	2.50	2.0 (∼1 m from patient)
8	1 ft from closed door waist level	0.20	–
9	1 ft from wall inside Hot Lab	0.75	0.30
10	1 m from unshielded source of radiation	4.00	–

^a^ All measurements performed using the same calibrated ionization chamber survey meter (Fluke 452, Fluke Biomedical).

For **T** **ask** **(4c.i.)**, the procedures will address how the areas will be prepared to prevent contamination by covering surfaces that come in contact with the drug, usually with absorbent disposable paper. This section will also include patient preparation procedures, both medically‐speaking and radiation‐wise. For Lu‐177‐PSMA‐617, patients can void immediately prior to infusion to minimize the need for restroom usage (and contamination) after administration. Radiation safety instructions during and post‐administration should be reviewed with the patient. Patients can be treated in the seated position, with an indwelling intravenous (IV) catheter placed in the arm, saline infusion of at least 10 ml to prime and check for patency of tubing/lines and to check for any swelling, pain, or discomfort at the injection site due to extravasation. Midline catheterization or peripherally inserted central catheters (PICCs) may serve as alternate approaches for infusion in difficult‐to‐access venous situations, and to reduce the risk of infiltration in select cases.^23^ Absorbent paper should be placed around the lines, and the injection site. For more complex infusions with greater chance of contamination, one may ask the patient to change into hospital gowns or disposable clothing prior to infusion. Staff involved with infusion should be wearing personal protective equipment (PPE) and personal radiation dosimeters. Geiger‐Muller (GM) survey meters should be readily available to check for any contamination of staff prior to leaving the restricted area. All of these steps can be performed prior to bringing the vial/syringe into the treatment room.

For **Task** **(4c.ii.)**, the therapy administration procedures will describe the infusion methods, which will need to be developed at the center. At the current time, Lu‐177‐PSMA‐617 is delivered to the center in a ready‐to‐use (i.e., radiolabeling is complete) single‐dose 30 ml vial (fill volume of ∼7‐12 ml) with an activity of 7.4 GBq (200 mCi) calibrated for date and time of treatment. The contents of the vial can be infused intravenously either directly from the vial at rates regulated by an infusion pump^24^ or by infusion of saline into the vial to force the contents out via the gravity infusion method (with or without an infusion pump), similar to what has been described previously for Lu‐177 DOTATATE treatments.^23^, ^25^ The gravity infusion method requires careful monitoring of infusion rates to avoid leakage resulting from pressure increases and overflow in the vial. Vial‐based infusion times are typically within 30 min. Alternatively, a more efficient delivery over a period of 1–10 min can be achieved via a syringe for infusion either with or without a syringe pump. However, the syringe infusion method does require additional preparatory steps of dispensation of the drug from the vial into the syringe (as described for **T** **asks** **4b.iii.–4b.iv**.). Figure 2 shows the treatment setup and administration for a manual syringe injection of Lu‐177‐PSMA‐617. With vial or syringe, there is a need to monitor for leaks and for extravasation during the infusion. The process for infusion should be developed and tested as part of commissioning, and may benefit from the use of checklists.^26^ Steps to address extravasation can also be included on the checklist, and should follow guidelines in place at the treating center. Some commonly recommended steps including pausing the infusion, elevating the injection site, and applying warm compresses on the infusion site to accelerate vasodilation and blood flow to the tissues allowing dispersion of the drug from the area. An example of additional actions on how to evaluate the dose to the injection site has been published elsewhere for Lu‐177 DOTATATE.^27^

For **Task** **(4** **c.iii.)**, post‐therapy procedures include determination of the administered activity, patient monitoring, patient release and radiation safety instruction, handling of certain situations after release, contamination checks, and area release. Methods are needed for determination of the administered activity, including assay of the vial/syringe and lines post‐infusion to assess any residual activity not delivered to the patient. Lu‐177‐PSMA‐617 therapy may involve monitoring the patient for up to 60 min with vital signs checked every 15 min. The key preparatory step here is to prepare the restroom for contamination if the patient needs to void during this monitoring time as the urine will be radioactive. Patient release should be evaluated per the criteria stated in 10 CFR 35.75, that is if the total effective dose equivalent to any other individual from exposure to the released individual is not likely to exceed 500 mrem. If satisfied, the treating center can avoid quartering of these patients as RAM inpatients (§35.315), avoiding the need for private hospital rooms and for radiation‐trained inpatient care staff (§35.310). It has been our experience that exposure levels from Lu‐177‐PSMA‐617 patients immediately after infusion are about 2 mR/h at 1 m, and making conservative assumptions of no biologic elimination and an occupancy factor of 1.0, the maximally exposed individual would receive less than 500 mrem at 1 m due to total physical decay of the drug. Thus, at our center, these patients would be considered releasable with radiation safety instructions per 10 CFR 35.75, facilitating outpatient therapy.^13^ Basic release instructions are available from the study sponsor/company for a 7.4 GBq administration of Lu‐177‐PSMA‐617.^11^ These instructions include maintaining at least 1 m distance from others, avoiding exposure to pregnant women or children, urinating in the seated position, double‐flushing of the toilet, and good hand hygiene. These instructions are to be followed for 2–3 days, with those instructions pertaining to patients who may come in contact with children and pregnant persons followed for at least a week and 2 weeks, respectively. The timeline recommended by the sponsor/company is tied to the pharmacokinetic properties of the drug. In the case of PSMA‐617, any non‐tumor bound Lu‐177‐PSMA‐617 has a low molecular weight and is rapidly cleared from the body, on average within 48 h.^3^, ^5^ However, it should be noted that the length of time for patients to follow these instructions is an area of debate with little published data as patients may be shedding radiation via other means, for example, tumor cell lysis.

In some instances, the sponsor/manufacturer‐provided release instructions may also be supplemented by the treating center (licensee) for special cases. The handling of these special cases will vary from center to center, and should consider regulatory requirements (which can vary by state) as well as radiation safety concerns. As such, the treating center should work with the RSO in the design of additional instructions. As an example of a special case at our center, patients with Foley catheters may be given instructions, in addition to that already mentioned, to empty the urine bag directly into the sanitary sewage system more immediately and exchange the bag after about 3 days, and repeat once more. As another example at our center, patients with incontinence pads may be given additional instructions to exchange pads as soon as soiled and to wipe the skin with flushable wipes. In both cases, patients are advised at our center to store the soiled items in a separate trash bag kept in an isolated location for about 2 months before disposal as regular trash. In general, though, it is preferred that patients immediately dispose of their solid waste in the normal municipal waste stream. This minimizes the exposure to members of the household, who might otherwise reside within 3–4 m of the waste with >50% occupancy. However, this recommendation has presented some challenges relating to the radiation monitoring equipment at municipal waste facilities, in which case the licensee may need to work with the state regulators and municipal waste workers to clearly establish this disposal as being appropriate.

**Task** **(** **4c.iii**.**)** can also include procedures for handling of other situations after drug administration, including medical emergencies, handling of radioactive lab samples (if any), and patient expiration soon after completion of administration. If the patient is releasable per 10 CFR 35.75 but not medically fit for discharge or has a medical emergency that requires care within an emergency department (ED), then the release instructions can be made available to the ED care staff with special emphasis on the need for “universal precautions” as defined by the Centers for Disease Control.^28^ Due to the clearance of Lu‐177‐PSMA‐617, universal precautions are typically advised for three days post infusion. Universal precautions are also appropriate for medical lab personnel handling radioactive specimens and samples, if any.^29^ For patients that have expired, NRC regulatory guide 8.39 revision 1 describes a general procedure for radioactive decedents (www.nrc.gov).

Immediately after completion of the radiopharmaceutical administration, contamination checks should be performed using a suitable radiation meter before any staff, equipment, and patient belongings leave the restricted areas. Once vacated, ambient surveys and contamination wipes of these areas should be done, and, if needed, decontamination cleaning measures should be carried out prior to release of these areas for normal routine use. The amount of removable radioactive material per 100 cm^2^ of surface area should be determined by wipes and assessing the level of radioactivity on the wipe with an appropriate instrument of known efficiency.^15^ At our center, we use the limit of 200 decays per minute (dpm) per 100 cm^2^ for an unrestricted area. For fixed contamination (i.e., not removable), radiation surveys are done to ensure that the exposure rate readings are indistinguishable from background levels. Otherwise, the areas will remain restricted until the criteria for release are satisfied.^15^

**Task** **(** **4d** **)** is to explicitly recognize that written procedure generation will also include forms that go along with these processes (refer to **T** **ask** **(** **4d)** in the Implementation Tasklist for examples of forms).

## 6 CONDUCT TRAINING

Those involved with the handling of patients and RAM should be trained on the written procedures, and receive safety instructions. Records of safety training should be maintained (§35.2310).^13^ For those involved with infusion, they can be trained via mock infusion sessions using non‐radioactive solution, for example, dyed saline to watch the flow of the “drug” through the IV line(s). Just before initiating therapy, a “kick‐off meeting” to summarize the overall workflow for the first patient may be warranted.

## 7 INITIATE AND MAINTAIN THERAPY

Once therapy is initiated, it is important to maintain the therapy by providing on‐going support, updating the procedures and forms, and storing treatment records, for example, the written directive (§35.40) and records of administered activity (§35.2063), radiation survey results (§35.2070), and patient release (§35.2075).^13^ Maintaining the therapy may also include modifying the SOP in response to potential problems or actual events, such as near‐misses or failures, by implementing process improvement approaches, for example, failure modes and effects analysis (FMEA) or root‐cause analysis (RCA), as demonstrated previously for other radiopharmaceutical therapies.^23^, ^30^

## 8 KEY RECOMMENDATIONS FOR IMPLEMENTING A LU‐177‐PSMA‐617 THERAPY PROGRAM

### 8.1 Treatment area/restroom

There is a need for a dedicated administration area with appropriate contamination control, exposure control, restricted access, and, because of patient monitoring post‐infusion, a nearby restroom with similar requirements. For contamination control, we recommend starting with a more conservative approach by over‐preparing surfaces, and then scaling back the amount of preparation with experience and comfort level.

### 8.2 Treatment administration

Efficient treatment (radiopharmaceutical) administration can be achieved via a syringe. However, at this time, the dosage is shipped in a vial, and, therefore, methods and expertise are needed for dispensation of the dosage from vial into syringe. Otherwise, the drug can be administered directly from the vial with generally longer treatment times and, if the gravity infusion method is used, may involve a greater risk of contamination. Use of checklists can provide added layers of safety during infusion. We recommend deciding on the infusion technique and practicing it via mock infusion sessions using dyed saline in place of radioactive drug.

### 8.3 Patient release

For a 7.4 GBq infusion, patients that are medically fit for discharge are also typically radiation‐releasable with instructions per 10 CFR 35.75, facilitating outpatient therapy.^13^ Instructions are readily available from the study sponsor/company and can be tailored with the help of the RSO for the release of special cases, for example, incontinent patients. If the patient is not medically fit for discharge after treatment, patient care staff may be required to handle inpatient scenarios under the guidance of universal precautions. Thus, we recommend that the procedure for release address both outpatient and inpatient situations.

### 8.4 RAM waste

Be aware of the production method for the isotope. There may be need for a DIS program that accommodates the disposal of long‐lived contaminant waste (Lu‐177m). We recommend working with the RSO at your center to design the RAM waste program.

### 8.5 Test drug shipment

We recommend using the calibration vial shipment as a dry‐run to validate the procedures and forms. Ensure that the equipment, draft of procedures/forms, and key staff are ready and available as this should be one of the last commissioning steps prior to initiating therapy.

### 8.6 Written procedures and training

We recommend investing the effort to develop and maintain a written operating procedure that can be useful as a training resource for everyone involved with the therapy to competently perform their duties and to safely handle RAM; all implemented in a manner that is in line with the regulations and best practice recommendations.^13^, ^14^, ^15^, ^16^

## AUTHOR CONTRIBUTION

All of the authors have contributed directly to the intellectual content of the manuscript.

## CONFLICT OF INTEREST

No relevant disclosures.
